# Treatment and re-operation rates in one thousand and three hundred tibial fractures from the Swedish Fracture Register

**DOI:** 10.1007/s00590-020-02751-x

**Published:** 2020-08-02

**Authors:** David Wennergren, Carl Bergdahl, Amanda Selse, Jan Ekelund, Mikael Sundfeldt, Michael Möller

**Affiliations:** 1 Institute of Clinical Sciences, Sahlgrenska Academy, University of Gothenburg, Gothenburg, Sweden; 2 Department of Orthopaedics, Sahlgrenska University Hospital, SE-413 45 Mölndal, Gothenburg, Sweden; 3 Centre of Registers, Western Healthcare Region, Gothenburg, Sweden

**Keywords:** Tibial fracture, Treatment, Re-operation, Fracture register

## Abstract

**Purpose:**

Approximately, 50 persons per 100,000 per year sustain a tibial fracture. There is, however, a lack of large cohort studies that describe the treatment and re-operation frequencies of tibial fractures. The aim of this study was to describe the treatment and re-operation rates of tibial fractures in all segments of the tibia.

**Methods:**

Data related to all patients aged 16 and above treated for tibial fractures (ICD-10 S82.10-31) at Sahlgrenska University Hospital in 2011–2015 were extracted from the Swedish Fracture Register. To make sure all re-operations were included in the study, the operation planning system was checked for all patients included in the study.

**Results:**

The study comprised 1371 tibial fractures − 712 proximal, 417 diaphyseal and 242 distal tibial fractures. Among the proximal and distal tibial fractures, plate fixation was the most commonly used surgical method, whereas among tibial shaft fractures, an intramedullary nail was the most commonly used surgical method. Almost 30% (29.8%) of all surgically treated tibial fractures underwent re-operation. Among proximal tibial fractures, 24.0% underwent re-operation; tibial shaft fractures 37.0% and distal tibial fractures 26.8%. Re-operations due to infection were more or less equally common in all segments (3.9–5.4%).

**Conclusion:**

This study describes the treatment and re-operation rates after tibial fractures in a cohort of 1371 tibial fractures at Sahlgrenska University Hospital during a period of 5 years. The study shows an overall re-operation rate of 29.8% for fractures in all segments of the tibia.

## Introduction

Approximately, 50 persons per 100,000 and year sustain a tibial fracture [1]. During the past 20 years, the treatment of tibial fractures has evolved. New opportunities with anatomic plates and modern intramedullary nails have been developed. There is, however, a lack of large cohort studies that describe the treatment and re-operation frequencies of tibial fractures in everyday practice.

A few recent studies of different aspects of specific types of tibial fracture have reported re-operation rates after the treatment of tibial fractures [2–6]. To the best of our knowledge, there is, however, no previous register-based study that describes the treatment and re-operation rates for fractures in all segments of the tibia.

Some randomised controlled trials of the treatment of tibial fractures have been performed [3, 7–9]. They often focus on specific topics, such as plate fixation versus intramedullary nailing or reamed versus non-reamed intramedullary nailing in certain fracture types with specific inclusion and exclusion criteria. The Swedish Fracture Register (SFR), on the other hand, prospectively collects data on all patients with all types of fracture, regardless of treatment [10, 11]. Several validity and epidemiological studies based on data from the SFR have been published [1, 12–21]. Register-based studies, such as studies based on data from the SFR, include all patients and fractures, regardless of treatment, and can describe the current treatment and results of the treatment algorithms being used in current clinical practice.

The aim of this study was to describe the treatment and re-operation rates of tibial fractures in all segments of the tibia for a cohort of consecutive tibial fractures at one large hospital over a period of 5 years.

## Materials and methods

Data related to all patients treated for tibial fractures (ICD-10 S82.10-31) at Sahlgrenska University Hospital in 2011–2015 were extracted from the SFR. This includes isolated tibial fractures as well as tibial fractures as part of multiply injured patients. Sahlgrenska University Hospital covers a population of approximately 530,000 inhabitants aged 16 and above in the primary catchment area and 1,700,000 inhabitants (all ages) in the secondary catchment area and is the only hospital in the area treating tibial fractures [1]. The study is based on the same cohort as a previous study on epidemiology and incidence of tibial fractures. Data such as mean age, range and distribution among fracture classes are described in detail in that publication [1]. The data extraction was performed in 2018 and the minimum follow-up period is 2 years (range 2–8 years). The data consist of information on the patient’s date of birth, the date and cause of the injury, high- or low-energy trauma, fracture classification according to AO/OTA, all treatments of each fracture, including primary treatments, planned secondary surgery and re-operations, as well as the reason for re-operation [22, 23]. Data on ligament injuries are not collected in the SFR.

All treatments performed for a fracture were registered in the SFR, i.e. primary treatments, planned secondary treatments and re-operations. Decision regarding treatment for each fracture was decided by the attending orthopaedic surgeon. Surgical as well as non-surgical treatments were registered. Non-surgical treatment included treatment with plaster, orthosis and fractures where no active treatment was given. When re-operations are performed, the re-operation together with the reason for the re-operation is registered, e.g. non-union, malunion, infection and patient discomfort. Patient discomfort was typically when internal fixation material, e.g. a locking screw in an intramedullary nail, caused the patient local pain. The design of the SFR and the registration process have been described in detail in two earlier publications [10, 11]. Like a previous study of epidemiology in the same cohort, the current study includes patients aged 16 and above [1]. At the Department of Orthopaedics, Sahlgrenska University Hospital there are more or less well defined traditions and practice of how different types of tibial fractures are treated. There are, however, no formal protocols concerning treatment choice. In clinical practice it is the surgeon’s choice how to treat the fracture at hand. The same applies to implant removal.

To make sure all re-operations were included in the study, the operation planning system was checked for all patients included in the study. If a treatment not registered in the SFR was detected, the medical chart was reviewed and missed treatments were registered in the SFR. Subsequently, a new data extraction was made from the SFR on which the calculations and analysis for the study were based.

## Statistical analysis

The study only contains descriptive statistics. No statistical tests between groups and no sample size calculation were therefore performed. All statistics for tables and figures in the study were calculated with IBM SPSS 25 and SAS v 9.4.

### Ethics

The study was approved by the Central Ethical Review Board, Gothenburg (Ref nr: 594–16).

### Human and animal rights

This is a register study and no Human and Animal Rights were violated.

## Results

### Treatment

**Fig. 1:**
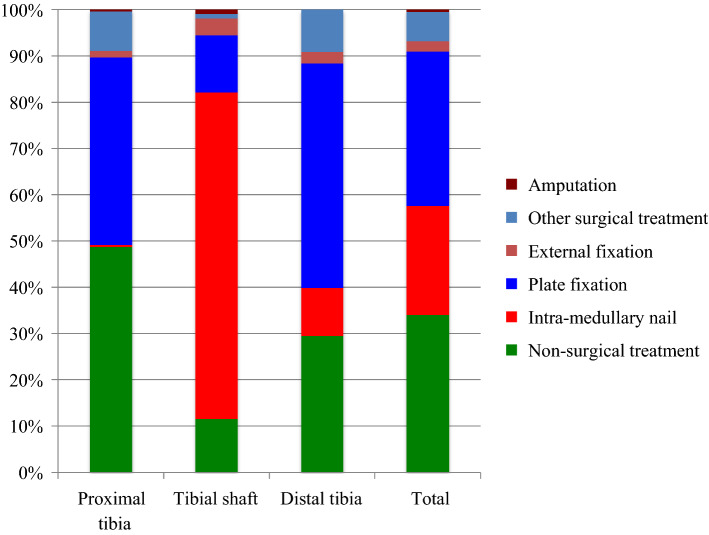
Distribution of treatment of tibial fractures according to segment of tibia

The study comprised 1371 tibial fractures − 712 proximal, 417 diaphyseal and 242 distal tibial fractures. The majority (66%) of tibial fractures were treated surgically, but 34% were treated non-surgically (Fig. 1). For all tibial fractures, 1672 surgical procedures were performed − 888 primary surgical procedures, 302 planned secondary procedures (e.g. staged procedures such as intramedullary nailing or plate fixation after temporary external fixation) and 463 re-operations. Nineteen tibial fractures were primarily assigned to non-surgical treatment, but, at an early stage, they were converted to surgical treatment after non-surgical treatment was considered inappropriate, e.g. due to a more severe dislocation found at an early x-ray check-up (“Surgical treatment after failed non-surgical treatment”) (Table 1).

**Table 1:** Number of treatments performed for all tibial fractures at Sahlgrenska University Hospital in 2011–2015

	Segment			
	Proximal (*N* = 712)	Shaft (*N* = 417)	Distal (*N* = 242)	Total (*N* = 1371)
Non-surgical treatment	352	62	75	489
Primary surgical treatment	358	361	169	888
Planned secondary procedure	97	83	122	302
Surgical treatment after failed non-surgical treatment*	7	10	2	19
Re-operation due to non-union	6	37	14	57
Re-operation due to malunion	29	17	12	58
Re-operation due to infection	32	20	14	66
Re-operation due to implant failure	4	22	12	38
Re-operation due to patient discomfort	42	62	14	118
Re-operation due to other reason	40	63	23	126

*Fractures that were primarily assigned to non-surgical treatment, but which at an early stage were converted to surgical treatment after non-surgical treatment was considered inappropriate

The completeness of the registration of treatments (the extent to which the treatments that were performed had been initially registered in the SFR) was 99.1% for primary procedures, 88.7% for planned secondary surgery and 63% for re-operations (Table 2).

**Table 2:** Number of initially missed registrations of procedures and completeness according to type of procedure for tibia fractures at Sahlgrenska University Hospital 2011–2015

	Missed registrations	Total number of procedures	Completeness (%)
Primary procedure	12	1396	99.1
Planned secondary surgery	34	302	88.7
Re-operation	171	462	63.0
Total	217	2160	90.0

Non-surgical treatment was chosen in 341/699 (49%) of proximal tibial fractures, 48/411 (12%) of tibial shaft fractures and 68/237 (29%) of distal tibial fractures (Table 3). Table 3 also shows that, among the proximal and distal tibial fractures, plate fixation is the most commonly used surgical method, whereas among tibial shaft fractures, intramedullary nailing is the most commonly used surgical method. A total of seven patients (0.5%) underwent amputation. Three of these were fractures that were not possible to classify, two were proximal and two were tibial shaft fractures. However, no distal tibial fractures underwent amputation. Table 4 shows that five of these amputations were performed as re-operations, whereas the remaining two amputations were performed as primary treatments.

**Table 3:** Treatment of tibial fractures according to AO/OTA class at Sahlgrenska University Hospital in 2011–2015

AO/OTA class	Non-surgical	IM nail	Plate fixation	External fixation	Other surgical treatment*	Amputation	Total
*Treatment, number of fractures (%)*							
41-A1	38 (64)	0 (0)	1 (2)	0 (0)	20 (34)	0 (0)	59
41-A2	27 (49)	1 (2)	26 (47)	1 (2)	0 (0)	0 (0)	55
41-A3	1 (8)	1 (8)	10 (83)	0 (0)	0 (0)	0 (0)	12
41-B1	119 (66)	1 (1)	45 (25)	0 (0)	15 (8)	0 (0)	180
41-B2	107 (77)	0 (0)	24 (17)	0 (0)	8 (6)	0 (0)	139
41-B3	37 (29)	0 (0)	81 (64)	0 (0)	8 (6)	0 (0)	126
41-C1	9 (20)	0 (0)	33 (73)	0 (0)	3 (7)	0 (0)	45
41-C2	1 (5)	0 (0)	14 (74)	2 (11)	2 (11)	0 (0)	19
41-C3	2 (3)	0 (0)	52 (81)	7 (11)	1 (2)	2 (3)	64
Total 41	341 (49)	3 (0.4)	286 (41)	10 (1)	57 (8)	2 (0.3)	699
42-A1	15 (13)	82 (73)	12 (11)	4 (4)	0 (0)	0 (0)	113
42-A2	6 (12)	38 (75)	3 (6)	1 (2)	2 (4)	1 (2)	51
42-A3	19 (29)	41 (62)	2 (3)	2 (3)	2 (3)	0 (0)	66
42-B1	4 (10)	31 (76)	5 (12)	1 (2)	0 (0)	0 (0)	41
42-B2	2 (4)	38 (76)	9 (18)	1 (2)	0 (0)	0 (0)	50
42-B3	1 (4)	14 (58)	6 (25)	2 (8)	0 (0)	1 (4)	24
42-C1	0 (0)	26 (84)	5 (16)	0 (0)	0 (0)	0 (0)	31
42-C2	0 (0)	14 (82)	2 (12)	1 (6)	0 (0)	0 (0)	17
42-C3	1 (6)	8 (44)	6 (33)	3 (17)	0 (0)	0 (0)	18
Total 42	48 (12)	292 (71)	50 (12)	15 (4)	4 (1)	2 (0.5)	411
43-A1	30 (54)	11 (20)	15 (27)	0 (0)	0 (0)	0 (0)	56
43-A2	2 (29)	1 (14)	4 (57)	0 (0)	0 (0)	0 (0)	7
43-A3	3 (10)	11 (37)	13 (43)	3 (10)	0 (0)	0 (0)	30
43-B1	29 (57)	0 (0)	8 (16)	1 (2)	13 (25)	0 (0)	51
43-B2	1 (8)	0 (0)	9 (69)	0 (0)	3 (23)	0 (0)	13
43-B3	1 (8)	0 (0)	9 (69)	0 (0)	3 (23)	0 (0)	13
43-C1	2 (22)	0 (0)	7 (78)	0 (0)	0 (0)	0 (0)	9
43-C2	0 (0)	2 (18)	8 (73)	1 (9)	0 (0)	0 (0)	11
43-C3	0 (0)	0 (0)	44 (94)	1 (2)	2 (4)	0 (0)	47
Total 43	68 (29)	25 (11)	117 (49)	6 (3)	21 (9)	0 (0)	237
Not able to classify	6 (50)	0 (0)	1 (8)	0 (0)	2 (17)	3 (25)	12
Total	463 (34)	320 (24)	454 (33)	31 (2)	84 (6)	7 (0.5)	1359

*“Other surgical treatment” includes screw fixation, pin fixation, fixation with cerclage, etc

The percentage figures refer to the percentage within each row, i.e. the percentage per AO/OTA class. 10 fractures had missing information regarding main treatment and two fractures were classified as paediatric fractures and are not included in this table. As a result, the total number of fractures in the table is 1359

**Table 4:** Number of re-operation procedures according to AO/OTA class and type of procedure based on all surgically treated fractures

AO/OTA	IM nail	Plate fixation	External fixation	Other fracture fixation*	Arthro-plasty	Arthro-desis	Amputation	Arthro-scopy	Correction osteotomy	Other soft- tissue surgery**	Removal of internal devices	Extraction of external fixation	Total number of procedures	Total number of reoperated fractures	Percentage reoperated fractures	Total number of surgically treated fractures
*Number of procedures*																
41-A1	0	0	0	0	0	0	0	2	0	2	0	0	4	4	19.0	21
41-A2	0	0	0	0	0	0	0	0	0	2	2	0	4	2	7.1	28
41-A3	0	0	0	0	0	0	0	0	0	1	1	0	2	2	18.2	11
41-B1	0	0	0	0	0	0	0	1	0	3	7	0	11	8	13.3	60
41-B2	0	0	0	0	2	0	0	0	1	0	8	0	11	9	28.1	32
41-B3	0	2	0	0	5	0	0	2	1	1	13	0	24	17	19.1	89
41-C1	0	1	1	1	0	0	0	4	1	3	8	0	19	10	27.8	36
41-C2	0	1	2	1	0	0	0	0	0	1	5	0	10	6	33.3	18
41-C3	0	1	1	1	7	1	2	4	2	15	26	1	61	29	46.0	63
Total 41	0	5	4	3	14	1	2	13	5	28	70	1	146	87	24.3	358
42-A1	4	2	0	4	0	0	0	0	0	0	31	0	41	29	29.6	98
42-A2	0	0	0	0	0	0	1	0	0	0	12	1	14	10	22.2	45
42-A3	2	0	0	2	0	0	0	0	0	0	27	0	31	21	47.7	44
42-B1	2	0	0	0	0	0	0	0	1	2	13	0	18	13	35.1	37
42-B2	3	1	4	1	0	0	0	0	1	4	22	2	38	22	45.8	48
42-B3	1	0	2	0	0	0	2	0	1	3	8	0	17	8	36.4	22
42-C1	3	1	0	1	0	0	0	0	0	2	19	0	26	16	51.6	31
42-C2	0	0	1	1	0	0	0	0	0	1	6	1	10	6	35.3	17
42-C3	1	2	0	2	0	0	0	0	1	2	7	0	15	8	47.1	17
Total 42	16	6	7	11	0	0	3	0	4	14	145	4	210	133	37.0	359
43-A1	0	1	2	0	0	0	0	0	0	1	5	0	9	6	25.0	24
43-A2	0	0	0	0	0	0	0	0	0	0	2	0	2	2	40.0	5
43-A3	2	0	3	2	0	0	0	0	0	0	8	0	15	9	33.3	27
43-B1	0	0	0	1	0	1	0	0	0	1	5	0	8	3	13.6	22
43-B2	0	0	0	1	0	0	0	0	0	0	3	0	4	3	25.0	12
43-B3	0	1	0	0	0	0	0	0	3	0	2	0	6	3	25.0	12
43-C1	0	0	0	0	0	0	0	0	0	0	1	0	1	1	14.3	7
43-C2	0	2	0	0	0	0	0	0	0	0	3	0	5	3	27.3	11
43-C3	0	2	2	2	0	3	0	0	0	6	14	1	30	14	29.8	47
Total 43	2	6	7	6	0	4	0	0	3	8	43	1	80	44	26.3	167
Not able to classify	0	0	0	1	0	0	0	0	0	1	0	0	2	1	16.7	6
Total	18	17	18	21	14	5	5	13	12	51	258	6	438	265	29.8	890

* “Other fracture fixation” includes screw fixation, pin fixation, fixation with cerclage and a combination of fixation methods

** “Other soft-tissue surgery” includes fasciotomy, surgical debridement and surgery due to superficial and deep infections, etc

Two fractures were classified as paediatric fractures and are not included in this table, so the total number of surgically treated fractures is 890. Please note that, in some cases, more than one procedure has been performed on one fracture and sometimes on one occasion. The total number of procedures is therefore higher than the total number of reoperated fractures. Table 4 presents all the procedures, regardless of whether they were performed in different re-operations or simultaneously in one re-operation. For example, if a patient undergoes surgical debridement and the removal of internal fixation devices in one re-operation, both these procedures are presented in Table 4

With regard to the proximal tibial fractures, the A1, A2, B1 and B2 fractures are the fractures most commonly treated non-surgically, whereas the A3, B3 and C fractures are most commonly treated surgically.

In terms of all fracture types among the tibial shaft fractures, the vast majority were treated surgically. Among all tibial shaft fractures, 71% were treated with intramedullary nailing. The A and B1 fractures were treated non-surgically in 10–29%, whereas the other fracture classes were almost exclusively treated surgically. Plate fixation was less common for A fractures, but it occurs in all fracture classes.

In terms of the distal tibia, among the A1 and B1 fractures, approximately half the fractures were treated non-surgically (54% and 57%, respectively). For all other distal tibial fractures, the majority were treated surgically, most commonly with plate fixation, apart from some A fractures that were treated with intramedullary nailing.

### Re-operations

29.8% of all tibial fractures underwent re-operation (Table 4). Among proximal tibial fractures, 24.0% underwent re-operation, tibial shaft fractures 37.0% and distal tibial fractures 26.8% (Table 5). The AO/OTA classes with the highest re-operation rates were 41C3 (46.0%), 42A3 (47.7%), 42B2 (45.8%), 42C1 (51.6%), 42C3 (47.1%) and 43A2 (40.0%) (Table 4). The removal of internal fixation devices is by far the most commonly performed re-operation (258/438 re-operations) (Table 4).

**Table 5:** Percentage reoperated fractures according to segment of the tibia and reason for re-operations based on all surgically treated fractures

	Non-union (%)	Malunion (%)	Infection (%)	Implant failure (%)	Patient discomfort (%)	Other reason (%)	Total re-operations (%)
Proximal tibia	0.6	4.7	3.9	0.8	9.1	9.1	24.0
Tibial shaft	5.2	2.2	4.1	3.6	15.7	14.1	37.0
Distal tibial	4.2	4.2	5.4	3.6	7.7	8.3	26.8
Total	3.1	3.6	4.3	2.5	11.5	11	29.8

Table 5 presents percentage reoperated fractures according to reasons for re-operations per segment of the tibia. Among the proximal tibial fractures, 0.6% of the surgically treated fractures underwent re-operation due to non-union and 4.7% due to malunion. Among the tibial shaft fractures, it was the other way around − 5.2% of the surgically treated fractures underwent re-operation due to non-union, whereas 2.2% due to malunion. Among the distal tibial fractures, re-operation due to non-union and malunion were equally common − 4.2% each. Re-operations due to infection were more or less equally common in all segments of the tibia (3.9%, 4.1% and 5.4%, respectively).

Tables 6, 7, and 8 present the number of re-operations according to reason for re-operation and main treatment for each segment of the tibia. Twenty-three per cent of the proximal tibial fractures treated with plate fixation underwent re-operation. For tibial shaft fractures, the re-operation rates were the same for fractures treated with intramedullary nailing and plate fixation (39% each) (Table 7). Among tibial shaft fractures treated with an intramedullary nail, 107 re-operations due to patient discomfort and “other reasons” were performed in 292 fractures, while, among tibial shaft fractures treated with plate fixation, ten re-operations due to patient discomfort and “other reasons” were performed in 51 fractures. Among the 51 tibial shaft fractures treated with plate fixation, seven re-operations were performed due to non-union, seven due to infection and five due to implant failure. Among the 292 tibial shaft fractures treated with an intramedullary nail, 19 re-operations were performed due to non-union, 11 due to infection and ten due to implant failure. Re-operations for malunion in tibial shaft fractures were only performed in fractures treated with an intramedullary nail.

**Table 6:** Number of re-operations according to main treatment and reason for re-operation for proximal tibia fractures

		Non-union	Malunion	Infection	Implant failure	Patient discomfort	Other reason	Total number of re-operations	Total number of reoperated fractures	Percentage reoperated fractures	Total number of fractures
*Reason for re-operation*											
Main treatment	Non-surgical	0	3	1	0	2	0	6	6	2	344
	IM nail	1	0	0	0	0	0	1	1	33	3
	Plate fixation	3	16	20	3	31	24	97	65	23	286
	External fixation	0	3	3	0	0	2	8	5	50	10
	Other surgical treatment*	0	1	0	0	6	8	15	14	23	60
	Amputation	0	0	1	0	0	2	3	2	67	3
Total		4	23	25	3	39	36	130	93	13	706

* “Other surgical treatment” includes screw fixation, pin fixation, fixation with cerclage, etc

Six proximal tibia fractures had missing information regarding main treatment, so the total number of fractures in the table is 706

**Table 7:** Number of re-operations according to main treatment and reason for re-operation for tibial shaft fractures

		Non-union	Malunion	Infection	Implant failure	Patient discomfort	Other reason	Total number of re-operations	Total number of reoperated fractures	Percentage reoperated fractures	Total number of fractures
*Reason for re-operation*											
Main treatment	Non-surgical	0	0	0	0	0	0	0	0	0	48
	IM nail	19	10	11	10	62	45	157	114	39	292
	Plate fixation	7	0	7	5	0	10	29	20	39	51
	External fixation	0	0	0	0	0	2	2	1	7	15
	Other surgical treatment*	0	0	0	0	0	0	0	0	0	4
	Amputation	0	0	2	0	0	3	5	3	75	4
Total		26	10	20	15	62	60	193	138	33	414

* “Other surgical treatment” includes screw fixation, pin fixation, fixation with cerclage, etc

Three tibial shaft fractures had missing information regarding main treatment, so the total number of fractures in the table is 414

**Fig. 2:**
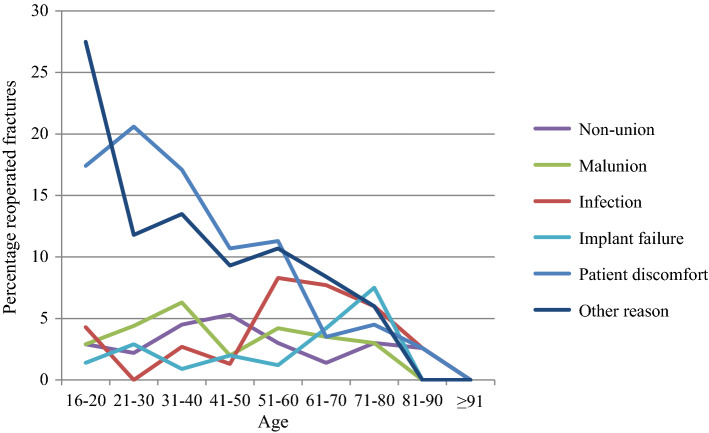
Percentage reoperated fractures according to reason for re-operation and age

Of the surgically treated tibial fractures, 3.1% underwent re-operation due non-union, 3.6% due to malunion, 4.3% due to infection and 2.5% due to implant failure (Table 5). The re-operation rates due to infection appear to be higher in patients 51–80 years of age (Fig. 2). For re-operations due to non-union, malunion and implant failure, however, there is no obvious difference in re-operation rates in the different age groups. Re-operations due to patient discomfort and other reasons appear to be more commonly performed in younger patients (age ≤ 60).

Of the 118 re-operations performed due to patient discomfort, 102 involved the removal of internal fixation devices. Of the 126 re-operations performed due to “other reasons”, 73 involved the removal of internal fixation devices.

## Discussion

### Treatment

The most important finding in terms of treatment is that 49% of proximal tibial fractures, 12% of tibial shaft fractures and 29% of distal tibial fractures were treated non-surgically. The most commonly used surgical method was plate fixation for proximal and distal tibial fractures and intramedullary nailing for tibial shaft fractures. For most of the AO/OTA fracture classes, more than 60% of the fractures were treated with one specific method (e.g. non-surgical treatment, an intramedullary nail or plate fixation).

In many ways, there were clear patterns of treatment for tibial fractures in the current study, according to the AO/OTA classification. When reviewing the specific fracture classes, in 10 of 27 fracture classes, more than 75% of the fractures were treated with one specific treatment method (e.g. non-surgical treatment, an intramedullary nail or plate fixation) and, in 20 of 27 fracture classes, more than 60% are treated with one specific treatment method. This can be interpreted as meaning that the treatment choice for tibial fractures is often not controversial. Another interesting finding was that, in all segments, the “1” and “2” fractures (e.g. 41A1 and A2, 41B1 and 2, 43A1 and 43B1) appear to be more commonly treated non-surgically, whereas the more complex “3” fractures appear to be treated surgically to a larger extent. This supports the idea that the AO/OTA classification system is predictive of treatment choice [22, 23].

### Re-operations

The most important finding in terms of re-operations is an overall re-operation rate (percentage reoperated fractures among the surgically treated fractures) of almost 30% (29.8%) for fractures in all segments of the tibia. Tibial shaft fractures had a higher re-operation rate (37.0%) than proximal and distal tibial fractures (24.0% and 26.8%, respectively). The removal of internal fixation devices was the most commonly performed re-operation (258 of a total of 438 re-operations). In proximal tibial fractures, re-operations due to non-union were less common than re-operations due to malunion (0.6% reoperated fractures versus 4.7%). This confirms the belief that metaphyseal bone often do not pose healing problems and non-union is uncommon. In tibial shaft fractures it was the other way around, re-operations due to non-union were more common than re-operations due to malunion (5.2% reoperated fractures versus 2.2%). This was also expected since cortical, diaphyseal bone often heals slower. In distal tibial fractures, re-operations due to non-union and malunion were equally common (4.2% reoperated fractures each). As discussed later in the context of infection this might be due to diminished blood supply to the distal end of the extremities. Otherwise one might expect fractures of the metaphyseal bone of the distal tibia to have a low frequency of non-union as in the proximal tibia.

Probably the largest published study on re-operation rates after tibial fractures is the study by Henry et al. that presents re-operation rates and mortality after tibial plateau fractures [6]. Although no specific classification of fractures is reported in the study by Henry et al., it is stated in the paper that tibial plateau fractures correspond to 41A-C fractures in the AO/OTA classification. Henry et al. showed that 15.3% of the patients with tibial plateau fractures underwent re-operations. In the current study, the corresponding figures for proximal tibial fractures are 24%. The higher numbers in the current study could be due to a longer follow-up period in the current study. In a prospective study of 275 consecutive surgically treated proximal tibial fractures, Kugelman et al. reported a higher risk of complications for AO/OTA C fractures [4]. These findings are in agreement with the findings in the current study, where there was a high frequency of reoperated fractures among the 41C fractures (27.8–46.0%).

In a systematic review, Henkelmann et al. report an infection rate of 9.8% (range 2.6–45.0%) in proximal tibial fractures [24]. In the current study, 3.9% of proximal tibial fractures underwent re-operation due to infection. It is, however, difficult to compare the numbers from the current study with the systematic review, as the current study is based on re-operations and the re-operation rates were not reported in the review. In the current study, there was a peak of re-operations due to infection at the age of 51–80 years (6–8.3% reoperated fractures). It is difficult to say what the cause of this might be. One possible explanation might be comorbidity, with diminished blood supply to the lower extremities. It is also possible that the soft-tissue injuries in this age group are underestimated.

Re-operations among tibial shaft fractures treated with an intramedullary nail and plate fixation in the current study were equally common (39% each). However, it appears that fractures treated with an intramedullary nail underwent re-operation to a greater extent due to patient discomfort or “other reasons” which, in most cases, was related to the removal of hardware, whereas the tibial shaft fractures treated with plate fixation underwent re-operation to a greater extent due to non-union, infection and implant failure, which are re-operations due to more severe complications. The higher rate of re-operations due to more severe complications among the tibial shaft fractures treated with plate fixation might be due to larger surgical exposures compared with the percutaneous intramedullary nailing. Even though removal of hardware is not a severe complication, to correctly inform patients and to plan the health care system, it is important to know that a large part of patients treated with an intramedullary nail subsequently undergoes removal of hardware.

As far as we can tell, there is no other study with the same design, which makes it difficult to compare the results with previous studies. Fong et al. described that 13.5% of tibial shaft fractures underwent re-operation (not including hardware removal), which is similar to the re-operation rate in the current study (if removal of hardware is excluded) [2]. Costa et al. found that osteosynthesis with an intramedullary nail and plate fixation in distal extra-articular tibial fractures had similar functional results [3]. In the study by Costa et al., more secondary operations and infections were found among the fractures treated with plate osteosynthesis compared with the fractures treated with an intramedullary nail. Costa et al. defined distal extra-articular fractures as “a fracture within two Müller squares of the ankle joint”. This renders a mixture of fractures that in the AO/OTA classification are classified as tibial shaft and distal tibial fractures, which makes it difficult to compare the results with the results of the current study. In the study by Minhas et al., there was no difference in re-operation rates between fractures treated with intramedullary nailing compared with plate fixation [5]. In the current study, the tibial shaft fractures treated with an intramedullary nailing and plate fixation showed overall re-operation rate of 39% each. The studies by Fong et al., Minhas et al. and the current study are all non-randomised studies where the treatment has been the responsible surgeon’s choice. Fong et al. reported no difference in re-operation rates and Minhas et al. and the current study report equal re-operation rates among patients with tibial shaft fractures that were treated with intramedullary nailing and plate fixation, respectively. One possible interpretation of this is that, in clinical practice, orthopaedic surgeons select the fractures that are best treated with intramedullary nailing and plate fixation, respectively.

When the SFR was started in 2011, there were fewer options available for registration of reasons for re-operations. After a few years it was, however, assumed that most of the re-operations registered as due to “other reasons” were in fact due to patient discomfort. Therefore, the possibility to register re-operations as due to “patient discomfort” was added in February 2016. Since 2016 registrations of re-operations due to “other reasons” have become much more uncommon. So, it can be assumed that the majority of re-operations registered as “due to other reasons” were in fact performed due to patient discomfort. In Tables 1, 5, 6, 7, 8 and Fig. 2, re-operations “due to other reasons” and “due to patient discomfort” have a similar distribution, which also supports this assumption. However, we claim that this does not affect the results of the study.

**Table 8:** Number of re-operations according to main treatment and reason for re-operation for distal tibia fractures

		Non-union	Malunion	Infection	Implant failure	Patient discomfort	Other reason	Total number of re-operations	Total number of reoperated fractures	Percentage reoperated fractures	Total number of fractures
*Reason for re-operation*											
Main treatment	Non-surgical	1	0	0	0	0	0	1	1	1	71
	IM nail	6	1	0	2	2	5	16	9	36	25
	Plate fixation	2	8	11	5	8	12	46	33	28	117
	External fixation	1	0	0	0	0	1	2	1	17	6
	Other surgical treatment*	1	0	2	1	3	2	9	4	18	22
	Amputation	0	0	0	0	0	0	0	0	0	0
Total		11	9	13	8	13	20	74	48	20	241

* “Other surgical treatment” includes screw fixation, pin fixation, fixation with cerclage, etc

One distal tibia fracture had missing information regarding main treatment, so the total number of fractures in the table is 241

The most obvious strength of the current study is that it presents a detailed description of the treatment and re-operation rates in a cohort of consecutive patients with tibial fractures during 5 years. The study includes all patients, all treatments, all types of fractures and all segments of the tibia. As the study does not exclude any type of patients, fractures or treatments, it describes the treatment and re-operation rates in a real-world setting. An additional strength is that Sahlgrenska University Hospital treats all tibial fractures in Gothenburg. The follow-up period of 2 to 8 years is comparatively long. Moreover, the classification of fractures for a sample of the cohort has been previously validated [12]. Thanks to the fact that the operation planning system and the medical charts were reviewed to ensure that all treatments including re-operations were registered in the SFR, high level of completeness was secured in the current study.

One limitation of the study is that it is based on a single hospital. The single centre design of the study was however a prerequisite for performing the above mentioned measures to achieve high completeness in registrations of both fractures, primary treatments and re-operations. Even though the single centre design is a limitation, the assurance of high completeness and validity of data in the study is a strength. Another limitation is that register-based data do not reveal every clinical aspect of the patients’ status and performance, such as pain, mobility, range of motion and radiographic healing.

The current study is based on register data and reveals how tibial fractures are treated in current clinical practice at one large hospital in Sweden. It describes the re-operation frequencies of different fracture types and different treatments in the whole of the tibia. This kind of data cannot be used to compare different treatments or to draw conclusions about which treatment is associated with the lowest re-operation frequency. Nevertheless, it describes the reality in a systematic and detailed way that has not been done before.

## Conclusions

This study describes the current treatment and re-operation rates of tibial fractures in Gothenburg, Sweden. For proximal and distal tibial fractures, plate fixation was the most commonly used surgical method and, for tibial shaft fractures, it was intramedullary nailing.

The study reveals an overall re-operation rate of 29.8% for fractures in all segments of the tibia. The re-operation rates described in the current study is important to be aware of to correctly inform patients and to plan the health care system.

## Data Availability

The datasets used and analysed during the current study are available from the corresponding author on reasonable request.

## Abbreviations

AO: Arbeitsgemeinschaft für osteosynthesefragen
OTA: Orthopaedic Trauma Association
SFR: Swedish Fracture Register
ICD-10: International Classification of Diseases Tenth Revision
